# Conformational Propensities of a DNA Hairpin with a Stem Sequence from the c-MYC Promoter

**DOI:** 10.3390/biom15040483

**Published:** 2025-03-26

**Authors:** Arees Garabet, Iztok Prislan, Nataša Poklar Ulrih, James W. Wells, Tigran V. Chalikian

**Affiliations:** 1 Department of Pharmaceutical Sciences, Leslie Dan Faculty of Pharmacy, University of Toronto, 144 College Street, Toronto, ON M5S 3M2, Canada; arees.garabet@utoronto.ca (A.G.); j.wells@utoronto.ca (J.W.W.); 2 Biotechnical Faculty, Department of Food Science and Technology, University of Ljubljana, Jamnikarjeva 101, 1000 Ljubljana, Slovenia; iztok.prislan@bf.uni-lj.si (I.P.); natasa.poklar@bf.uni-lj.si (N.P.U.)

**Keywords:** DNA, G-quadruplex, *i*-motif, hairpin duplex, conformational states, circular dichroism, thermodynamics, biophysical chemistry

## Abstract

G-quadruplexes and *i*-motifs are four-stranded non-canonical structures of DNA. They exist in the cell, where they are implicated in the conformational regulation of cellular events, such as transcription, translation, DNA replication, telomere homeostasis, and genomic instability. Formation of the G-quadruplex and *i*-motif conformations in the genome is controlled by their competition with the pre-existing duplex. The fate of that competition depends upon the relative stabilities of the competing conformations, leading ultimately to a distribution of double helical, tetrahelical, and coiled conformations that coexist in dynamic equilibrium with each other. We previously developed a CD spectroscopy-based procedure to characterize the distribution of conformations adopted by equimolar mixtures of complementary G- and C-rich DNA strands from the promoter regions of the c-MYC, VEGF, and Bcl-2 oncogenes. In those bimolecular systems, duplex-to-tetraplex and duplex-to-coil transitions are accompanied by strand separation and an associated entropic cost. This situation is distinct from the pseudo-monomolecular nature of conformational transformations within the genome, where strand separation does not occur. To mimic better the situation in the genome, we here extend our studies to a monomolecular DNA construct—a hairpin—in which complementary G- and C-rich strands featuring sequences from the promoter region of the c-MYC oncogene are linked by a dT_11_ loop. We used our CD-based procedure to quantify the distribution of conformational states sampled by the hairpin at pH 5.0 and 7.0 as a function of temperature and the concentration of KCl. The data were analyzed according to a thermodynamic model based on equilibria between the different conformational states to evaluate the thermodynamic properties of the duplex-to-coil, G-quadruplex-to-coil, and *i*-motif-to-coil transitions of the hairpin. The results have implications for the modulation of such transitions as a means of therapeutic intervention.

## 1. Introduction

G-quadruplexes and *i*-motifs are non-canonical four-stranded DNA structures that exist in the cell and have been implicated in the conformational control of key cellular pathways and genomic events [1,2,3,4,5,6,7,8,9,10,11,12,13,14,15,16,17]. In the human genome, consensus G- and C-rich DNA sequences potentially capable of adopting G-quadruplex and *i*-motif structures have been found in hundreds of thousands of critically important loci, such as the promoter regions of oncogenes [2,18,19,20,21,22,23,24,25,26,27,28,29,30,31,32]. G-quadruplex-mediated regulatory pathways are involved in various processes, including telomere homeostasis, gene transcription and translation, and DNA replication [33,34,35]. In a recent example, it has been shown how an intricate conformational interplay among duplex, G-quadruplex, and *i*-motif conformations may determine the pathways and attendant structural transformations that ensue upon strand invasion of genomic G-quadruplex domains [36].

G-quadruplex sequence motifs have been found in about 40% of the promoters of human genes [18]. They are more prevalent in proto-oncogenes and regulatory genes than in housekeeping and tumor-suppressor genes [18,37,38]. Stable *i*-motif structures initially were thought to form only at slightly acidic pH, but they subsequently have been detected at neutral pH and visualized in the nuclei of live cells [11,13,39,40,41]. Both G-quadruplexes and *i*-motifs have been visualized and quantified throughout the MCF-7 cell cycle [42].

Any regulatory role of G-quadruplexes and *i*-motifs in genomic DNA is conditional upon their ability to compete with the duplex conformation and to form in a timely manner within the constraints of Watson–Crick base pairing. It follows that attempts to identify the sites and mechanisms of tetraplex-related effects depend critically upon an understanding of the kinetics and thermodynamics of duplex–tetraplex interconversions in the genome, particularly as a function of nucleotide sequence and environmental conditions [1,43]. Such knowledge also is needed for designing G-quadruplex- and *i*-motif-based nanodevices that respond to ions and pH [44,45,46].

In recognition of this need, we have undertaken studies into the conformational propensities of complementary G- and C-rich DNA sequences from the promoter regions of human oncogenes [47,48]. The different conformations adopted under different conditions are identified and quantified by means of CD spectroscopy [47,48]. Our results to date have revealed that equimolar mixtures of complementary strands from the promoter regions of the c-MYC, VEGF, and Bcl-2 oncogenes may exist as a mixture of the duplex, G-quadruplex, *i*-motif, and coiled conformations, with the fractional population of each determined by the pH, concentration of potassium ions, and temperature [47,48]. These findings were confirmed subsequently by others using NMR spectroscopy, which showed that DNA from the c-MYC promoter folds into the duplex and G-quadruplex structures at physiological pH and into the G-quadruplex and *i*-motif structures under slightly acidic conditions [49].

In the studies described above, the transition between the duplex conformation and any of the single-stranded conformations (G-quadruplex, *i*-motif, coil) was bimolecular and, as such, involved the physical separation (dissociation) of hybridized strands. A bimolecular duplex-to-single-strand transition is accompanied by a favorable change in translational entropy that skews the conformational distribution in a concentration-dependent manner. The bimolecular nature of such transitions contrasts with the pseudo-monomolecular nature of duplex–tetraplex interconversions in the genome, which are not accompanied by separation of the interacting strands.

**Scheme 1:**
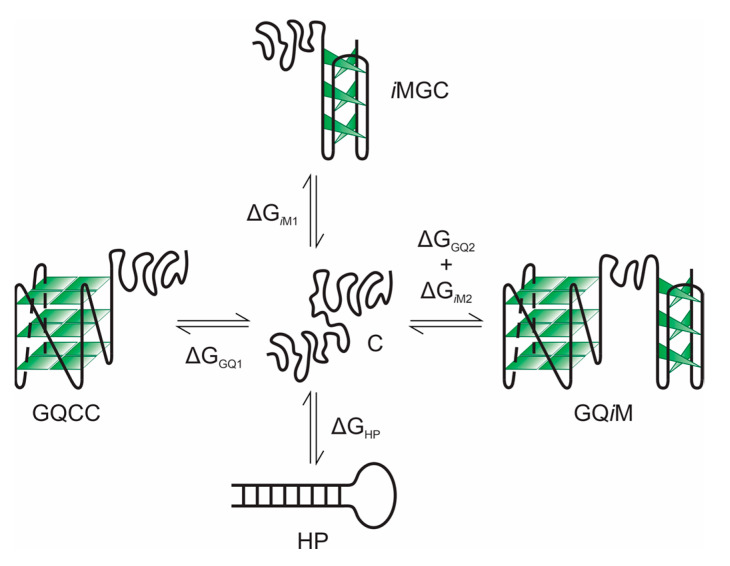
Equilibria and the changes in free energy linking the various states adopted by GT_11_C (C, coil; HP, hairpin; GQCC, G-quadruplex-plus-C-coil; iMGC, *i*-motif-plus-G-coil; GQiM, G-quadruplex-plus-*i*-motif). Tetraplex-containing states depicted in the model differ in their complement of G-quadruplex (GQ), *i*-motif (iM), and coil (GC, CC) conformations.

To address this difference, we report here a study of a hairpin-based monomolecular DNA construct (GT_11_C) in which a 22-base-pair stem is derived from the promoter region of the c-MYC oncogene [i.e., d(TGAGGGTGGGTAGGGTGGGTAA)/d(TTACCCACCCTACCCACCCTCA)]. The complementary G- and C-rich strands of the stem are linked via a dT_11_ loop. The G-rich d(TGAGGGTGGGTAGGGTGGGTAA) sequence is known to fold into a single-topology, parallel G-quadruplex in the presence of K^+^ ions [50]. Its complementary d(TTACCCACCCTACCCACCCTCA) sequence folds into an *i*-motif at ~pH 5.0 [51]. The construct has the potential to form a monomolecular “duplex”, or hairpin, and four different combinations of G-quadruplex, *i*-motif, and coil, as illustrated in Scheme 1. The complementary strands will dissociate but remain linked upon unfolding of the hairpin, and the system, therefore, mimics the pseudo-monomolecular situation in the genome.

The distribution of interconverting conformational states adopted by the hairpin DNA at equilibrium was characterized by CD spectroscopy in a manner described previously [47]. The procedure presupposes that the observed CD spectrum of a DNA in question is the weighted sum of the spectra of the constituent duplex, G-quadruplex, *i*-motif, and coiled conformations. Each spectrum therefore can be unmixed (deconvoluted) in terms of the predetermined spectra of the constituent conformational states to obtain the corresponding weighting factors for their contributions to the total population of DNA [47]. In the work described here, this CD-based approach has been used to determine the conformational states sampled by GT_11_C as a function of temperature and the concentration of potassium ions at neutral and slightly acidic pH (i.e., pH 7.0 and 5.0).

## 2. Materials and Methods

### 2.1. Materials

The oligonucleotides d(TGAGGGTGGGTAGGGTGGGTAA-T_11_-TTACCCACCCTACCCACCCTCA) (GT_11_C), dT_11_ (T_11_), d(TGAGGGTGGGTAGGGTGGGTAA-T_11_) (GT_11_), and d(T_11_-TTACCCACCCTACCCACCCTCA) (T_11_C) were purchased from Integrated DNA Technologies (Coralville, IA, USA). They were dissolved in 10 mM CsCl, dialyzed exhaustively against distilled water in Tube-O-Dialyzers (2000-Da cut-off, G Biosciences, St. Louis, MO, USA), and lyophilized. The lyophilized DNA then was dissolved in an appropriate experimental buffer and used without further dialysis. The concentrations of the oligonucleotides were determined spectrophotometrically. The molar extinction coefficients, ε_260_, were 508,000, 89,700, 317,1000, and 280,600 M^−1^cm^−1^ for the unfolded states of the d(TGAGGGTGGGTAGGGTGGGTAA-T_11_-TTACCCACCCTACCCACCCTCA), dT_11_, d(TGAGGGTGGGTAGGGTGGGTAA-T_11_), and d(T_11_-TTACCCACCCTACCCACCCTCA) strands, respectively, as computed using a nearest-neighbor procedure [52].

### 2.2. Circular Dichroism Spectropolarimetry

All CD spectra were recorded in a cuvette with a path-length of 1 mm using a JASCO J-1100 Circular Dichroism Spectrophotometer (JASCO, Easton, MD, USA). Measurements were conducted at pH 5.0 and 7.0. The pH 5.0 buffer consisted of 10 mM tetrabutylammonium (TBA^+^) acetate, while the pH 7.0 buffer consisted of 10 mM cesium phosphate or TBA^+^ phosphate. The buffers were supplemented with KCl or CsCl as required. TBA^+^ cations have been used extensively in studies of G-quadruplexes. Owing to their bulkiness, they can engage only in external interactions with G-quadruplexes. In contrast to small inorganic cations such as Na^+^ and K^+^, TBA^+^ cations cannot affect the stability of a G-quadruplex by penetrating its central cavity.

Temperature-dependent CD spectral measurements were conducted by decreasing the temperature from 95 to 25 °C in steps of 10 °C. The spectrum at each temperature was taken after equilibration of the sample for ten minutes. The concentrations of GT_11_C, GT_11_, T_11_C, and T_11_ in the cuvette were on the order of 20, 30, 30, and 90 μM, respectively.

### 2.3. UV Spectrophotometry

The concentrations of DNA were determined prior to the CD measurements via UV absorption at 260 nm. Spectrophotometric measurements were performed with a Cary 300 Bio spectrophotometer (Varian Canada, Inc., Mississauga, ON, Canada) in a cuvette with a path-length of 1 cm.

### 2.4. Deconvolution of CD Spectra

GT_11_C was assumed to interconvert among five different conformational states, as illustrated in Scheme 1: namely, coil (C), hairpin duplex (HP), G-quadruplex-plus-C-coil (GQCC), *i*-motif-plus-G-coil (iMGC), and G-quadruplex-plus-*i*-motif (GQiM). The populated states were quantified by means of a procedure in which the observed CD spectrum is taken as the weighted sum of the spectra of the constituent conformational states [47]; thus, the spectrum of GT_11_C in each conformational state is taken as the spectral sum of the T_11_ loop and the structures formed by the G-rich and C-rich strands of the stem. The CD spectrum of the coiled (C) state is equal to the sum of the CD spectra of the T_11_ loop (CD_T11_), the coiled G-rich strand (CD_GC_), and the coiled C-rich strand (CD_CC_). The CD spectrum of the HP state is equal to the sum of CD_T11_ and the CD spectrum of the duplex formed by the stem (CD_D_). The CD spectrum of the GQCC state is equal to the sum of CD_T11_, the CD spectrum of the G-quadruplex formed by the G-rich strand (CD_GQ_), and CD_CC_. The CD spectrum of the iMGC state is equal to the sum of CD_T11_, the CD spectrum of the *i*-motif formed by the C-rich strand (CD_iM_), and CD_GC_. Finally, the CD spectrum of the GQiM state is equal to the sum of CD_T11_, CD_GQ_, and CD_iM_.

With these assumptions, one arrives at a linear equation for partitioning the observed CD spectrum of GT_11_C into its weighted conformational components:

CD_obsd_ = α_HP_(CD_T11_ + CD_D_) + α_GQCC_(CD_T11_ + CD_GQ_ + CD_CC_) + α_iMGC_(CD_T11_ + CD_iM_ + CD_GC_) + α_GQiM_(CD_T11_ + CD_GQ_ + CD_iM_) + α_C_(CD_T11_ + CD_GC_ + CD_CC_) (1)

Since α_C_ + α_HP_ + α_GQCC_ + α_iMGC_ + α_GQiM_ = 1, one obtains:

CD_obsd_ = CD_T11_ + α_HP_CD_D_ + α_GQCC_(CD_GQ_ + CD_CC_) + α_iMGC_(CD_iM_ + CD_GC_) + α_GQiM_(CD_GQ_ + CD_iM_) + α_C_(CD_GC_ + CD_CC_) = CD_T11_ + α_HP_CD_D_ + (α_GQCC_ + α_GQiM_)CD_GQ_ + (α_iMGC_ + α_GQiM_)CD_iM_ + (α_iMGC_ + α_C_)CD_GC_ + (α_GQCC_ + α_C_)CD_CC_ (2)

Equation (2) can be rewritten as:

CD_obsd_ = CD_T11_ + F_HP_CD_D_ + F_GQ_CD_GQ_ + F_iM_CD_iM_ + F_GC_CD_GC_ + F_CC_CD_CC_ (3)

where F_HP_ = α_HP_ is the fraction of G- or C-strand in the hairpin duplex conformation; F_GQ_ = α_GQCC_ + α_GQiM_ and F_GC_ = α_iMGC_ + α_C_ are the fractions of G-strand in the G-quadruplex and coil conformations, respectively; and F_iM_ = α_iMGC_ + α_GQiM_ and F_CC_ = α_GQCC_ + α_C_ are the fractions of C-strand in the *i*-motif and C-coil conformations, respectively.

Since the fractions of each strand sum to 1 (i.e., F_HP_ + F_GQ_ + F_GC_ = 1 and F_HP_ + F_iM_ + F_CC_ = 1), one arrives at the following:

CD_obsd_ = CD_T11_ + F_HP_CD_D_ + F_GQ_CD_GQ_ + F_iM_CD_iM_ + (1 − F_HP_ − F_GQ_)CD_GC_ + (1 − F_HP_ − F_iM_)CD_CC_ = CD_T11_ + F_HP_(CD_D_ − CD_GC_ − CD_CC_) + F_GQ_(CD_GQ_ − CD_GC_)+ F_iM_(CD_iM_ − CD_CC_) + CD_GC_ + CD_CC_ (4)

Rearranging Equation (4) yields a relationship that can be used to deconvolute the experimental spectra of GT_11_C in terms of the fractional contributions of the constituent spectral components and their contributions to the total population of DNA:

CD_obsd_ − CD_T11_ − CD_GC_ − CD_CC_ = F_HP_(CD_D_ − CD_GC_ − CD_CC_) + F_GQ_(CD_GQ_ − CD_GC_) + F_iM_(CD_iM_ − CD_CC_) (5)

CD spectra were measured at wavelengths between 200 and 320 nm, with a step-size of 1 nm. Equation (5), therefore, transforms into an overdetermined system of 121 linear equations with three unknowns: F_HP_, F_GQ_, and F_iM_. We solved this system in MATLAB (v. R2022b) (The Mathworks, Inc., Natick, MA, USA) to evaluate the fractional populations, F_HP_, F_GQ_, F_iM_, F_GC_ = 1 − F_HP_ − F_GQ_, and F_CC_ = 1 − F_HP_ − F_iM_.

## 3. Results

### 3.1. CD Spectra of GT_11_C

**Figure 1:**
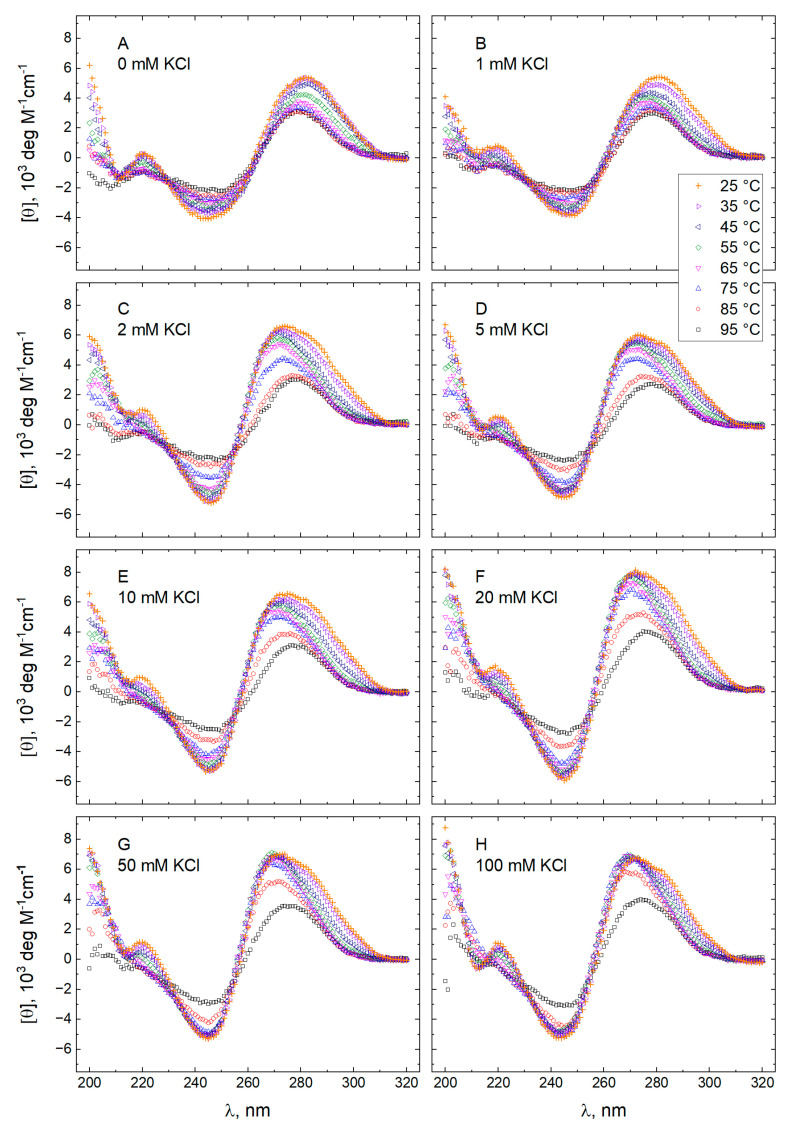
Molar CD spectra of GT_11_C at pH 5.0. Spectra were recorded at temperatures between 25 and 95 °C in a 10 mM TBA^+^ acetate buffer containing KCl at the following concentrations (mM): 0 (**panel A**), 1 (**panel B**), 2 (**panel C**), 5 (**panel D**), 10 (**panel E**), 20 (**panel F**), 50 (**panel G**), and 100 (**panel H**).

**Figure 2:**
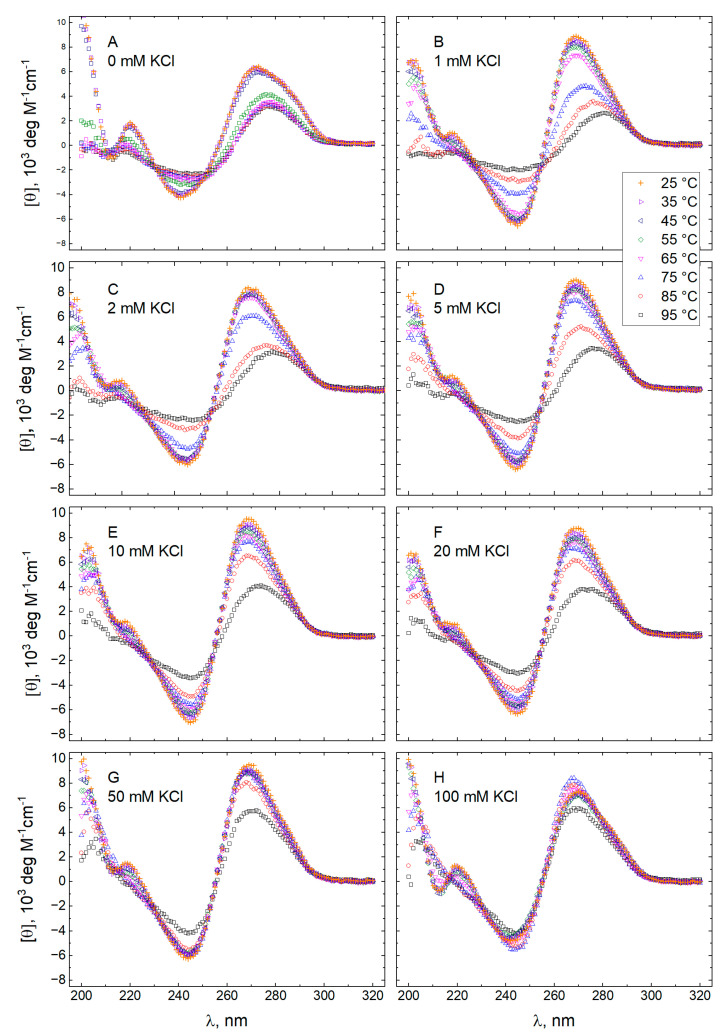
Molar CD spectra of GT_11_C at pH 7.0. Spectra were recorded at temperatures between 25 and 95 °C in a 10 mM TBA^+^ phosphate buffer acetate buffer containing KCl at the following concentrations (mM): 0 (**panel A**), 1 (**panel B**), 2 (**panel C**), 5 (**panel D**), 10 (**panel E**), 20 (**panel F**), 50 (**panel G**), and 100 (**panel H**).

**Figure 3:**
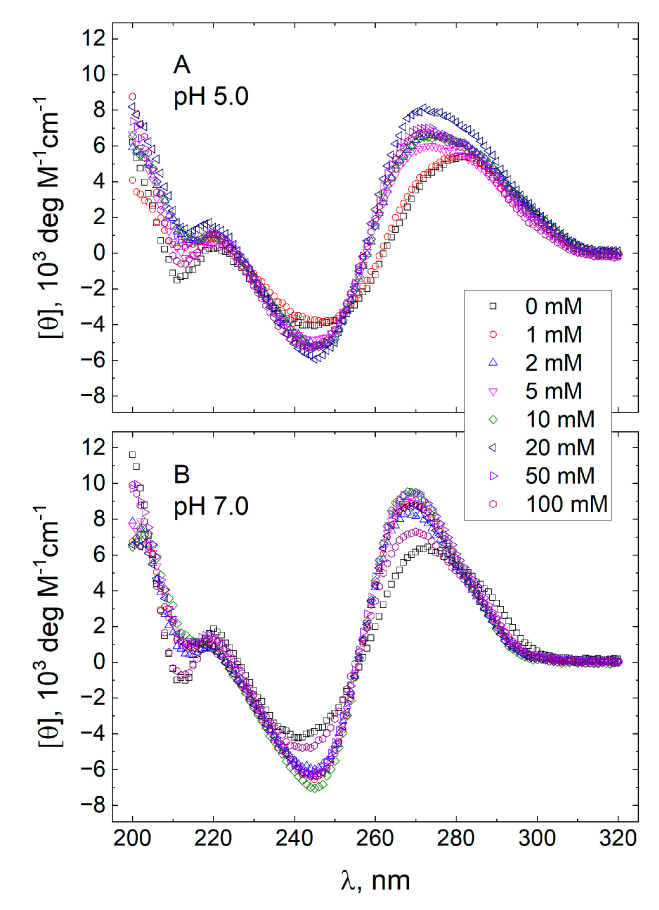
Molar CD spectra of GT_11_C at pH pH 5.0 (**panel A**) and pH 7.0 (**panel B**). Spectra were recorded at 25 °C in a 10 mM TBA^+^ acetate buffer (**panel A**) or a 10 mM TBA^+^ phosphate buffer (**panel B**) containing KCl at concentrations of 0, 1, 2, 5, 10, 20, 50, and 100 mM, as shown in the inset.

The molar CD spectra of GT_11_C in 10 mM TBA^+^ acetate buffer adjusted to pH 5.0 and in 10 mM TBA^+^ phosphate buffer adjusted to pH 7.0 are shown in Figure 1 and Figure 2, respectively. In each case, the spectra were recorded at eight concentrations of KCl from 0 to 100 mM (panels A–H) at temperatures descending from 95 to 25 °C. Spectra obtained at 25 °C and different concentrations of KCl are compared in Figure 3, where an increase in the concentration of KCl can be seen to effect pronounced changes in the CD spectrum of GT_11_C at pH 5.0 (Figure 3A) and pH 7.0 (Figure 3B). Such changes are consistent with a potassium-induced formation of G-quadruplex by the G-rich strand. This conclusion is supported by the melting profile of GT_11_C at pH 7.0 and 10 mM KCl, as recorded at 295 nm, which is the hallmark wavelength for G-quadruplex melting [53,54].

**Figure 4:**
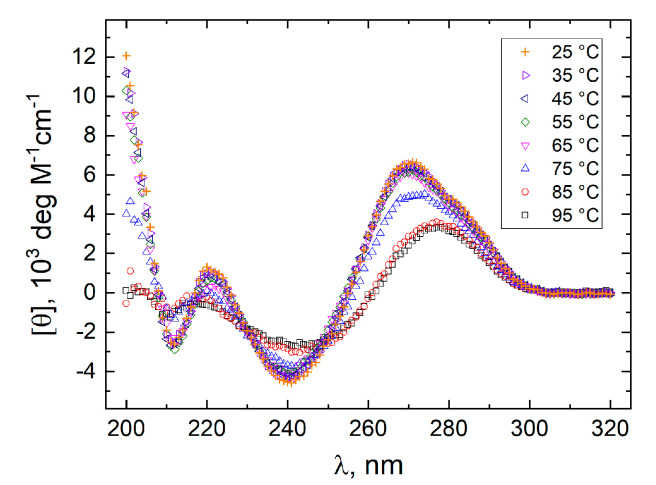
Molar CD spectra of GT_11_C at pH 7.0. Spectra were recorded at temperatures between 25 and 95 °C in a 10 mM cesium phosphate buffer containing 20 mM KCl.

CD spectra of GT_11_C recorded at different temperatures in the absence of TBA^+^ ions are shown in Figure 4, where the data were obtained at pH 7.0 in 10 mM cesium phosphate buffer containing 20 mM KCl. The spectra in Figure 4 are nearly identical to those recorded at pH 7.0 in TBA^+^ phosphate buffer but in the absence of KCl (Figure 2A). The implications of this similarity are considered below.

### 3.2. CD Spectra of “Pure” Conformations

The deconvolution of an observed CD spectrum according to Equation (5) requires the CD spectra of the “pure” duplex (CD_D_), G-quadruplex (CD_GQ_), *i*-motif (CD_iM_), G-coil (CD_GC_), and C-coil (CD_CC_) conformations. Those spectra were inferred from comparative analyses of the molar CD spectra of the GT_11_C, T_11_, GT_11_, and T_11_C oligonucleotides, which were recorded under conditions that were chosen to favor the conformation of interest while ensuring the virtual absence of other conformations. The determination presupposes that an observed spectrum is the weighted sum of its component parts.

**Figure 5:**
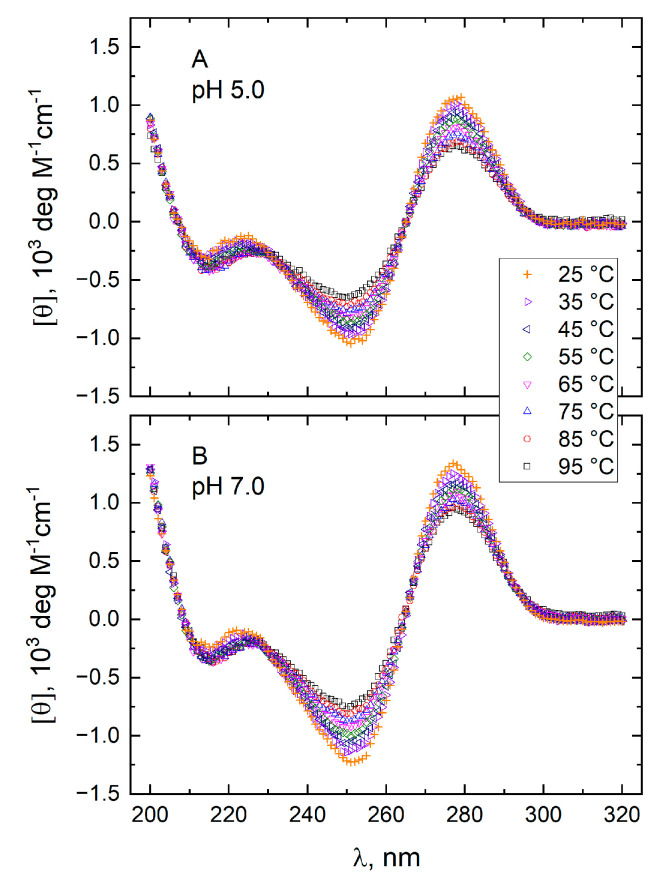
Molar CD spectra of T_11_ at pH 5.0 in a 10 mM TBA^+^ acetate buffer (**panel A**) and at pH 7.0 in a 10 mM TBA phosphate buffer (**panel B**). Spectra were recorded at temperatures between 25 and 95 °C in the absence of KCl.

**Figure 6:**
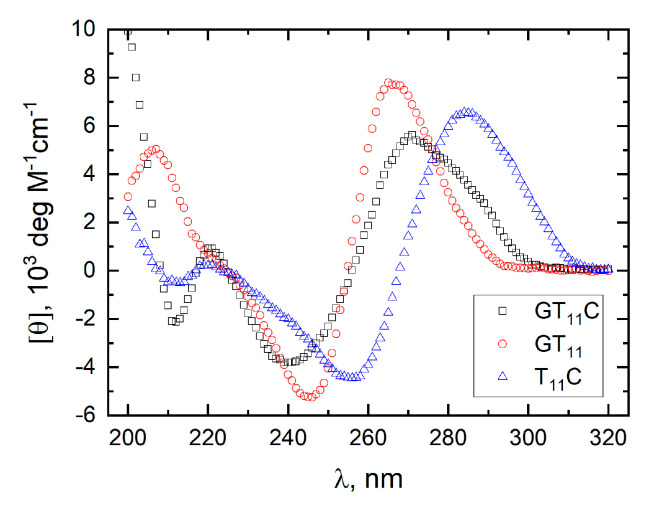
Molar CD spectra of GT_11_C at pH 7.0 in a 10 mM TBA^+^ phosphate buffer containing 10 mM CsCl (black, ☐), of GT_11_ at pH 7.0 in a 10 mM TBA^+^ phosphate buffer containing 5 mM KCl (red, ⭘), and of T_11_C at pH 5.0 in a 10 mM TBA^+^ acetate buffer lacking KCl (blue, △). All spectra were recorded at 25 °C.

**Figure 7:**
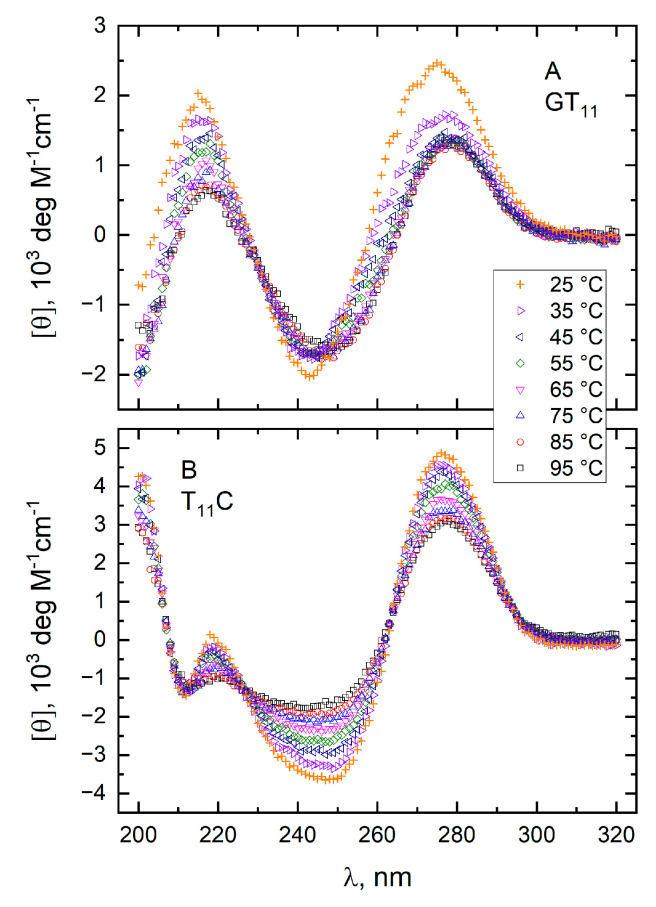
Molar CD spectra of GT_11_ (**panel A**) and T_11_C (**panel B**) at pH 7.0. Spectra were recorded at temperatures between 25 and 95 °C in a 10 mM TBA^+^ phosphate buffer in the absence of KCl.

The molar CD spectra of T_11_ at temperatures between 25 and 95 °C are shown in Figure 5, either in a 10 mM TBA^+^ acetate buffer adjusted to pH 5.0 (panel A) or in a 10 mM TBA^+^ phosphate buffer adjusted to pH 7.0 (panel B). The molar CD spectra of the other three oligonucleotides (GT_11_C, GT_11_, and T_11_C), all recorded at 25 °C, are shown in Figure 6 as follows: (i) GT_11_C (black, ☐) under conditions that favor the hairpin duplex while precluding the G-quadruplex and *i*-motif (i.e., a 10 mM TBA^+^ phosphate buffer adjusted to pH 7.0 and containing 10 mM CsCl but no KCl); (ii) GT_11_ (red, ⭘) under conditions that favor a G-quadruplex in the d(TGAGGGTGGGTAGGGTGGGTAA) domain and an unstructured dT_11_ domain (i.e., a 10 mM TBA^+^ phosphate buffer adjusted to pH 7.0 and containing 5 mM KCl); (iii) T_11_C (blue, △) under conditions that favor an *i*-motif in the d(TTACCCACCCTACCCACCCTCA) domain and an unstructured dT_11_ portion (i.e., a 10 mM TBA^+^ acetate buffer adjusted to pH 5.0). The molar CD spectra of GT_11_ and T_11_C at temperatures between 25 and 95 °C are shown in Figure 7A and Figure 7B, respectively, where the spectra were recorded in a 10 mM TBA^+^ phosphate buffer adjusted to pH 7.0 and lacking KCl. Under those conditions, both GT_11_ and T_11_C are unfolded over the entire temperature range; accordingly, the CD spectra in Figure 7A and Figure 7B can be used to extract the pure spectra of the G-coil and C-coil conformations, respectively (see below).

**Figure 8:**
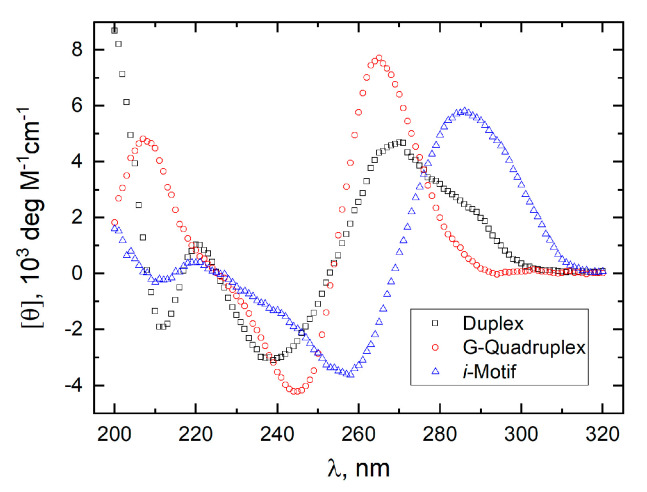
Molar CD spectra of the “pure” duplex (black, ☐), G-quadruplex (red, ⭘), and *i*-motif (blue, △) conformations. The spectra were computed by assuming additivity as described in the text.

The CD spectrum of the “pure” duplex formed by the stem of GT_11_C (CD_D_) was computed as the difference at 25 °C between the spectrum of GT_11_C taken at pH 7.0 and 10 mM CsCl (Figure 6) and the spectrum of T_11_ taken at pH 7.0 (Figure 5B). Under those conditions, GT_11_C exists overwhelmingly if not entirely in the hairpin duplex state; T_11_ is unfolded throughout, although the degree of unfolding is temperature-dependent. The CD spectrum of the “pure” G-quadruplex formed by the G-strand of GT_11_C (CD_GQ_) was computed as the difference at 25 °C between the spectrum of GT_11_ at pH 7.0 and 5 mM KCl (Figure 6) and that of T_11_ at pH 7.0 (Figure 5B). The CD spectrum of the “pure” *i*-motif formed by the C-strand of GT_11_C (CD_iM_) was computed as the difference at pH 5.0 and 25 °C between the spectrum of T_11_C (Figure 6) and that of T_11_ (Figure 5A). Cations have little effect on the formation of *i*-motif [8,55]. The CD spectra obtained in this manner for the “pure” duplex (CD_D_), G-quadruplex (CD_GQ_), and *i*-motif (CD_iM_) conformations are shown in Figure 8. Note that CD_GQ_ exhibits a positive maximum at 264 nm and a negative minimum at 244 nm, which are characteristic of the parallel G-quadruplex topology [56,57,58].

**Figure 9:**
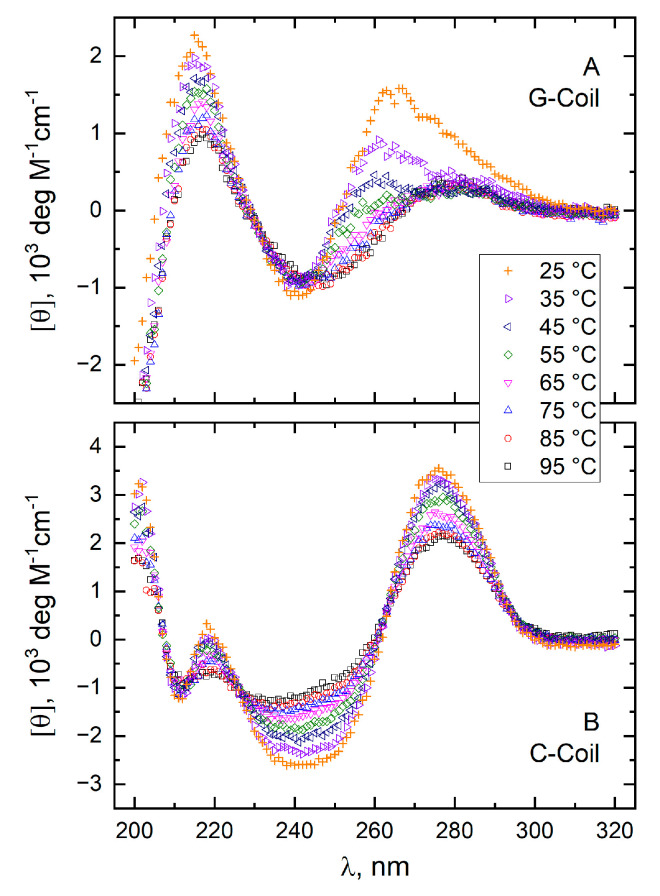
Molar CD spectra of the “pure” G-coil (**panel A**) and C-coil (**panel B**) conformations at temperatures between 25 and 95 °C. The spectra were computed by assuming additivity as described in the text.

In contrast to the CD spectra of the folded conformations (duplex, G-quadruplex, and *i*-motif), the spectra of the coiled conformations (G-coil and C-coil) are highly sensitive to temperature [47,48]. To obtain the temperature-dependent CD spectra of the unfolded G-coil and C-coil conformations, the spectrum of T_11_ at each temperature in Figure 5A,B was subtracted from the spectrum of the unfolded conformations of GT_11_ and T_11_C at the same temperature in Figure 7A and Figure 7B, respectively. The CD spectra obtained in this manner for the G-coil (CD_GC_) and C-coil (CD_CC_) at temperatures between 25 and 95 °C are shown in panels A and B, respectively, of Figure 9.

We previously have studied the conformational equilibria of a bimolecular system comprising the d(TGAGGGTGGGTAGGGTGGGTAA) and d(TTACCCACCCTACCCACCCTCA) DNA strands that constitute the two limbs of GT_11_C [47]. Because the two strands were separate molecular entities, the CD spectra of the duplex, G-quadruplex, *i*-motif, and coiled (G-coil and C-coil) conformations could be recorded directly in an assumption-free manner [47]. A comparison of the “pure conformation” CD spectra shown in Figure 8 and Figure 9, which were obtained by assuming that spectra are additive, with the corresponding, directly measured spectra from the earlier study [47], reveals that the two spectral sets are nearly identical. Because the DNA sequences studied previously were the same as the stem sequences of GT_11_C, the observed similarity argues for the validity of the assumptions and experimental protocols employed in the present study. It also suggests that the presence of the T_11_ loop in GT_11_C does not have an appreciable effect on the structural properties of the stem and vice versa.

### 3.3. Fractions of Individual Conformations 

**Figure 10:**
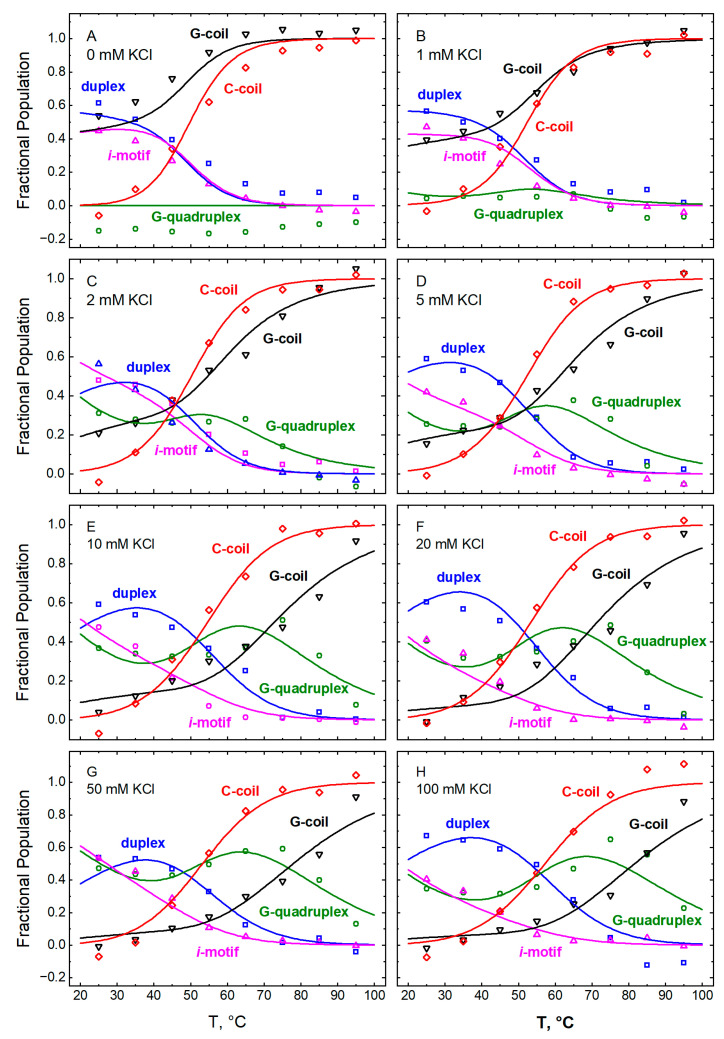
Temperature dependences of the fractional populations of the different conformations within GT_11_C at pH 5.0: hairpin duplex (blue, ☐), G-quadruplex (green, ⭘), *i*-motif (magenta, △), G-coil (black, ▽), and C-coil (red, ◇). The fractions were obtained by deconvolution of spectra recorded at the following concentrations of KCl (mM): 0 (**panel A**), 1 (**panel B**), 2 (**panel C**), 5 (**panel D**), 10 (**panel E**), 20 (**panel F**), 50 (**panel G**), and (**panel H**). The lines represent the best fit of Equations (8a)–(8e) to the data at all concentrations of KCl taken together. The fitted parametric values are listed in Table 1 and Table 2.

**Figure 11:**
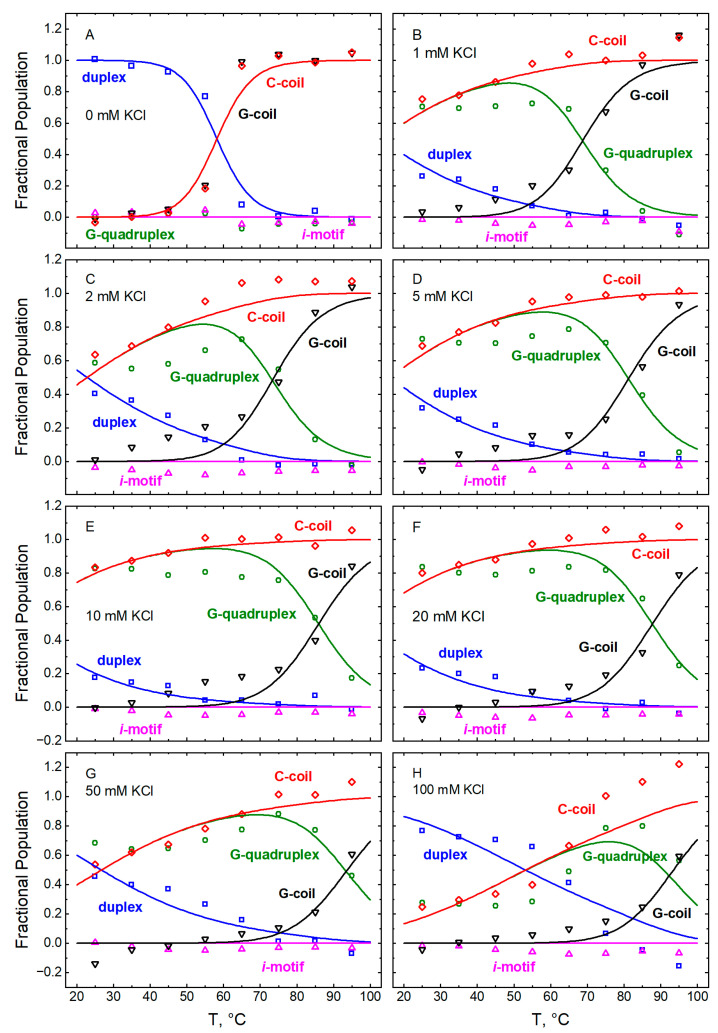
Temperature dependences of the fractional populations of the different conformations within GT_11_C at pH 7.0: hairpin duplex (blue, ☐), G-quadruplex (green, ⭘), *i*-motif (magenta, △), G-coil (black, ▽), and C-coil (red, ◇). The fractions were obtained by deconvolution of spectra recorded at the following concentrations of KCl (mM): 0 (**panel A**), 1 (**panel B**), 2 (**panel C**), 5 (**panel D**), 10 (**panel E**), 20 (**panel F**), 50 (**panel G**), and (**panel H**). The lines represent the best fit of Equations (8a)–(8e) to the data at all concentrations of KCl taken together. The fitted parametric values are listed in Table 1 and Table 3. In panel A, the fitted line for the G-coil obscures that for the C-coil; similarly, the fitted line for the *i*-motif obscures that for the G-quadruplex.

The CD spectra of GT_11_C recorded at pH 5.0 (Figure 1) and pH 7.0 (Figure 2) were used in conjunction with Equation (5) and the CD spectra of the “pure” conformations (Figure 8 and Figure 9) to compute the fractional populations of hairpin duplex (F_HP_), G-quadruplex (F_GQ_), *i*-motif (F_iM_), G-coil (F_GC_), and C-coil (F_CC_) at each temperature and each concentration of KCl. The computed values of F are plotted versus temperature in Figure 10 (pH 5.0) and Figure 11 (pH 7.0), where the data are grouped according to the concentration of KCl (panels A–H). The lines in each panel depict the best fit of the model represented by Scheme 1 and described below.

## 4. Discussion

### 4.1. Effect of TBA^+^ Ions on the Duplex-G-Quadruplex Competition

It is instructive to compare the CD spectra of GT_11_C obtained in three buffers at room temperature (25 °C) and pH 7.0: 10 mM TBA^+^ phosphate and 20 mM KCl (Figure 2F), 10 mM cesium phosphate and 20 mM KCl (Figure 4), and 10 mM TBA^+^ phosphate in the absence of KCl (Figure 2A). The first two spectra differ markedly despite the presence of 20 mM potassium ions in each case (cf. Figure 2F and Figure 4). In contrast, the spectrum at 25 °C in Figure 4 is nearly identical to that in Figure 2A, where there is no G-quadruplex and the hairpin exists overwhelmingly in the duplex conformation. We conclude from these comparisons that the presence of TBA^+^ ions is necessary for GT_11_C to form a G-quadruplex when in competition with a Watson-Crick duplex. In the absence of TBA^+^ ions, GT_11_C exists exclusively as a hairpin duplex, even at elevated concentrations of potassium ions; in the presence of TBA^+^ ions, 80% of the GT_11_C molecules form G-quadruplexes, and only 20% exist in the hairpin duplex conformation (see Figure 11F).

It is noteworthy that TBA^+^ ions are essential to the formation of a G-quadruplex but apparently are not required for an *i*-motif. In a TBA^+^-free cesium acetate buffer at pH 5.0 and 25 °C, GT_11_C exists as a mixture of the HP and iMGC states; there is no G-quadruplex, even at concentrations of KCl as high as 20 mM. These observations are consistent with our recent demonstration that tetraalkylammonium ions strongly stabilize parallel G-quadruplexes [59].

### 4.2. Thermodynamic Model

To determine the thermodynamic properties of conformational interconversions undergone by GT_11_C, the temperature dependences of the fractional populations plotted in Figure 10 and Figure 11 were analyzed according to a statistical thermodynamic model of the equilibria depicted in Scheme 1. The model presupposes that GT_11_C interconverts spontaneously among five conformational states: namely, the coil state (C), the hairpin state (HP), the double-tetraplex G-quadruplex-plus-*i*-motif state (GQiM), and the single-tetraplex G-quadruplex-plus-C-coil (GQCC) and *i*-motif-plus-G-coil (iMGC) states. The coil state is the ground state, and the free energies of all other states, ΔG, are computed relative to that of the ground state. The analyses were structured to optimize the values of the enthalpy (ΔH) and the melting temperature (T_M_) for each interconversion; those values were used in turn to compute the corresponding free energies, as described below.

It is assumed in the model that the free energy of tetraplex formation by the G-rich d(TGAGGGTGGGTAGGGTGGGTAA) strand of the stem depends upon the conformation of the C-rich d(TTACCCACCCTACCCACCCTCA) strand and vice versa. At pH 5.0, the G-rich strand may form a G-quadruplex in the presence or absence of an *i*-motif formed by the C-rich strand (i.e., the GQiM state or the GQCC state, respectively). In the same manner, the C-rich strand may form an *i*-motif in the presence or absence of a G-quadruplex formed by the G-rich strand (i.e., the GQiM state or the iMGC state, respectively). When formed together, the G-quadruplex and *i*-motif may interact with thermodynamic consequences.

Such an interaction manifests itself thermodynamically as an attendant change in free energy. This can be accommodated in our model as an additional contribution to the folding free energy of either the G-quadruplex formed by the G-strand or the *i*-motif formed by the C-strand when the two tetraplex conformations coexist in GT_11_C. We therefore assigned two separate free energy functions to the formation of the G-quadruplex: one for that in the absence of the *i*-motif (i.e., the GQCC state, ΔG_GQ1_) and another for that in the presence of the *i*-motif (i.e., the GQiM state, ΔG_GQ2_). An alternative arrangement would involve a single free energy function for the formation of the G-quadruplex and separate functions for that of the *i*-motif in the absence (i.e., the iMGC state) and in the presence (i.e., the GQiM state) of the G-quadruplex. The system is symmetrical with respect to the formation of the double tetraplex (GQ*i*M) from either single tetraplex (GQCC or *i*MGC), and the two approaches are mathematically and computationally equivalent.

In the model as formulated (Equations (6) and (7) below), the optimized parameters are the transition enthalpy (ΔH) and the melting temperature (T_M_). Preliminary analyses of the data at pH 5.0 confirmed that the fit was significantly better with two values of **Δ**H and T_M_ rather than one for either the G-quadruplex or the *i*-motif (*p* < 0.00001); no further reduction was obtained with two values of those parameters for both the G-quadruplex and the *i*-motif. The thermodynamic consequences of a tetraplex-tetraplex interaction within GQiM were assigned to the G-quadruplex, resulting in two values of **Δ**H_GQ_ and T_GQ_ for GQ (i.e., ΔH_GQ1_, ΔH_GQ2_, T_GQ1_, and T_GQ2_). The folding thermodynamics of the *i*-motif in the absence or presence of the G-quadruplex were assumed to be the same, and the parameters were optimized accordingly (i.e., ΔH_iM_ = ΔH_iM1_ = ΔH_iM2_, and T_iM_ = T_iM1_ = T_iM2_). These considerations apply exclusively to pH 5.0. At pH 7.0, the population of *i*-motif-containing molecules of GT_11_C is negligible.

For a monomolecular construct such as GT_11_C, the fractional populations of the individual states, α, in a system at equilibrium can be found from the following:

(6a) $$\alpha_{C}=\frac{1}{Q}$$

(6b) $$\alpha_{HP}=\frac{e^{-\frac{∆G_{HP}}{RT}}}{Q}$$

(6c) $$\alpha_{GQCC}=\frac{e^{-\frac{∆G_{GQ1}}{RT}}}{Q}$$

(6d) $$\alpha_{iMGC}=\frac{e^{-\frac{∆G_{iM}}{RT}}}{Q}$$

(6e) $$\alpha_{GQiM}=\frac{e^{-\frac{∆G_{GQiM}}{RT}}}{Q}=\frac{e^{-\frac{∆G_{GQ2}}{RT}}e^{-\frac{∆G_{iM}}{RT}}}{Q}$$

where the values of ΔG are the differences in free energy between the fully folded (HP, GQiM) or semi-folded (GQCC, iMGC) states and the coiled state (C), and $Q=1+e^{-\frac{∆G_{HP}}{RT}}+e^{-\frac{∆G_{GQ1}}{RT}}+e^{-\frac{∆G_{iM}}{RT}}+e^{-\frac{∆G_{GQiM}}{RT}}$ is the partition function.

The differential free energies of the folded states of GT_11_C and the ground state are given in turn by the following relationships:

(7a) $${∆G}_{HP}={∆H}_{HP}\left(1-\frac{T}{T_{HP}}\right)+{∆C}_{PHP}[T-T_{HP}-T\ln(\frac{T}{T_{HP}})]$$

(7b) $${∆G}_{GQ1}={∆H}_{GQ1}\left(1-\frac{T}{T_{GQ1}}\right)+{∆C}_{PGQ1}[T-T_{GQ1}-T\ln(\frac{T}{T_{GQ1}})]$$

(7c) $${∆G}_{GQ2}={∆H}_{GQ2}\left(1-\frac{T}{T_{GQ2}}\right)+{∆C}_{PGQ2}[T-T_{GQ2}-T\ln(\frac{T}{T_{GQ2}})]$$

(7d) $${∆G}_{iM}={∆H}_{iM}\left(1-\frac{T}{T_{iM}}\right)+{∆C}_{PiM}[T-T_{iM}-T\ln(\frac{T}{T_{iM}})]$$

(7e) $${∆G}_{GQiM}={∆G}_{GQ2}+{∆G}_{iM}={∆H}_{GQ2}\left(1-\frac{T}{T_{GQ2}}\right)+{∆H}_{iM}\left(1-\frac{T}{T_{iM}}\right)+\;{∆C}_{PGQ2}[T-T_{GQ2}-T\ln(\frac{T}{T_{GQ2}})]+{∆C}_{PiM}[T-T_{iM}-T\ln(\frac{T}{T_{iM}})]$$

where T_HP_, T_GQ1_, T_GQ2_, and T_iM_ are the helix-to-coil transition temperatures of the hairpin duplex, the G-quadruplex in the absence (T_GQ1_) and presence (T_GQ2_) of *i*-motif, and the *i*-motif, respectively; ΔH_HP_, ΔH_GQ1_, ΔH_GQ2_, and ΔH_iM_ are the changes in enthalpy accompanying the melting transitions of the hairpin duplex, the G-quadruplex in the absence (ΔH_GQ1_) and presence (ΔH_GQ2_) of *i*-motif, and the *i*-motif, each at its transition temperature; and ΔC_PHP_, ΔC_PGQ1_, ΔC_PGQ2_, and ΔC_PiM_ are the corresponding changes in heat capacity.

The transition temperatures in Equations (7a)–(7e) correspond to the temperatures at which the fractional population of the corresponding folded duplex or tetraplex conformation equals that of the unfolded coiled conformation, α_C_. For example, T_HP_ corresponds to the temperature at which the fractional population of the hairpin duplex state, α_HP_, equals α_C_. If only two states exist at equilibrium (i.e., the coiled state and one of the folded states), the transition temperature corresponds to the point of maximum slope (∂α/∂T) in the plot of fractional population versus temperature; otherwise, the transition temperature will differ from the temperature of maximum slope to a degree that depends upon the thermodynamic properties and the prevalence of additional states.

Combining Equations (6) and (7), one obtains the following relationships for the fractional populations of the duplex, F_HP_, G-quadruplex, F_GQ_, *i*-motif, F_iM_, G-coil, F_GC_, and C-coil, F_CC_, conformations:

(8a) $$F_{HP}=\alpha_{HP}=\frac{e^{-\frac{∆G_{HP}}{RT}}}{Q}$$

(8b) $$F_{GQ}=\alpha_{GQCC}+\alpha_{GQiM}=\frac{e^{-\frac{∆G_{GQ1}}{RT}}+e^{-\frac{∆G_{GQiM}}{RT}}}{Q}$$

(8c) $$F_{iM}=\alpha_{iMGC}+\alpha_{GQiM}=\frac{e^{-\frac{∆G_{iM}}{RT}}+e^{-\frac{∆G_{GQiM}}{RT}}}{Q}$$

(8d) $$F_{GC}=\alpha_{C}+\alpha_{iMGC}=\frac{1+e^{-\frac{∆G_{iM}}{RT}}}{Q}$$

(8e) $$F_{CC}=\alpha_{C}+\alpha_{GQCC}=\frac{1+e^{-\frac{∆G_{GQ1}}{RT}}}{Q}$$

### 4.3. Interpretation of the Temperature Dependences of Fractional Populations

The temperature dependences of the fractional populations of the duplex, G-quadruplex, *i*-motif, G-coil, and C-coil conformations were analyzed according to Equations (8a)–(8e) in conjunction with Equations (7a)–(7e). Data acquired at pH 5.0 (Figure 10A–H) and 7.0 (Figure 11A–H) were treated separately, with the 40 sets of data at each pH taken in concert (i.e., five conformations and eight concentrations of KCl).

It was assumed, as previously [47,48], that the transition enthalpies of the duplex (ΔH_HP_), G-quadruplex (ΔH_GQ1_ and ΔH_GQ2_), and *i*-motif (ΔH_iM_) conformations are independent of the concentration of KCl; accordingly, single values of those parameters were assigned to the data at all concentrations of KCl. This assumption implies that enthalpies do not depend upon the transition temperature, which is modulated by the concentration of KCl. The temperature dependences of enthalpies (ΔC_P_) therefore can be taken as zero, and all values of ΔC_P_ were fixed accordingly throughout the fitting procedure. It follows that salt-dependent variations in the transition free energies (i.e., ΔG_HP_, ΔG_GQCC_, ΔG_iMGC_, and ΔG_GQiM_) arise wholly from variations in the corresponding transition temperatures (i.e., T_HP_, T_GQ1_ and T_GQ2_, and T_iM_), which, therefore, were estimated separately for each concentration of KCl.

The fitted curves from the analyses are depicted by the lines in Figure 10 and Figure 11. The corresponding enthalpies at pH 5.0 and 7.0 are listed in Table 1 (ΔH_HP_, ΔH_GQ1_, ΔH_GQ2_, and ΔH_iM_); the transition temperatures at all concentrations of KCl are listed in Table 2 for pH 5.0 and in Table 3 for pH 7.0 (T_HP_, T_GQ1_, T_GQ2_, and T_iM_). Further details of the fitting procedure and a discussion of the possible sources of error have been given previously [47].

**Figure 12:**
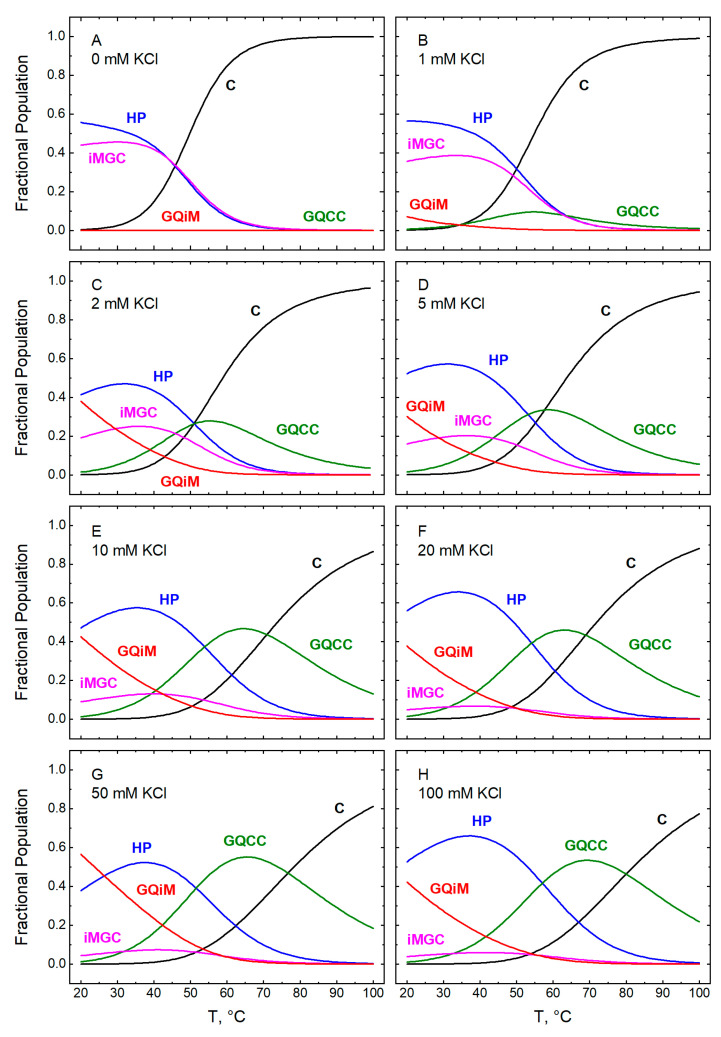
Temperature dependences computed for the fractional populations of the five conformational states of GT_11_C (Scheme 1) at pH 5.0: C (black), HP (blue), GQCC (green), iMGC (magenta), and GQiM (red). The fractions were calculated according to Equations (6a)–(6e) with the parametric values listed in Table 1 and Table 2 for the following concentrations of KCl (mM): 0 (**panel A**), 1 (**panel B**), 2 (**panel C**), 5 (**panel D**), 10 (**panel E**), 20 (**panel F**), 50 (**panel G**), and (**panel H**). In panel A, the line for GQiM obscures that for GQCC. Note that the term HP refers to the hairpin state, whereas the term duplex in Figure 10 refers to a secondary structure or conformation. Because the duplex exists only in the hairpin state, the corresponding curves in Figure 10 and Figure 12 are identical.

**Figure 13:**
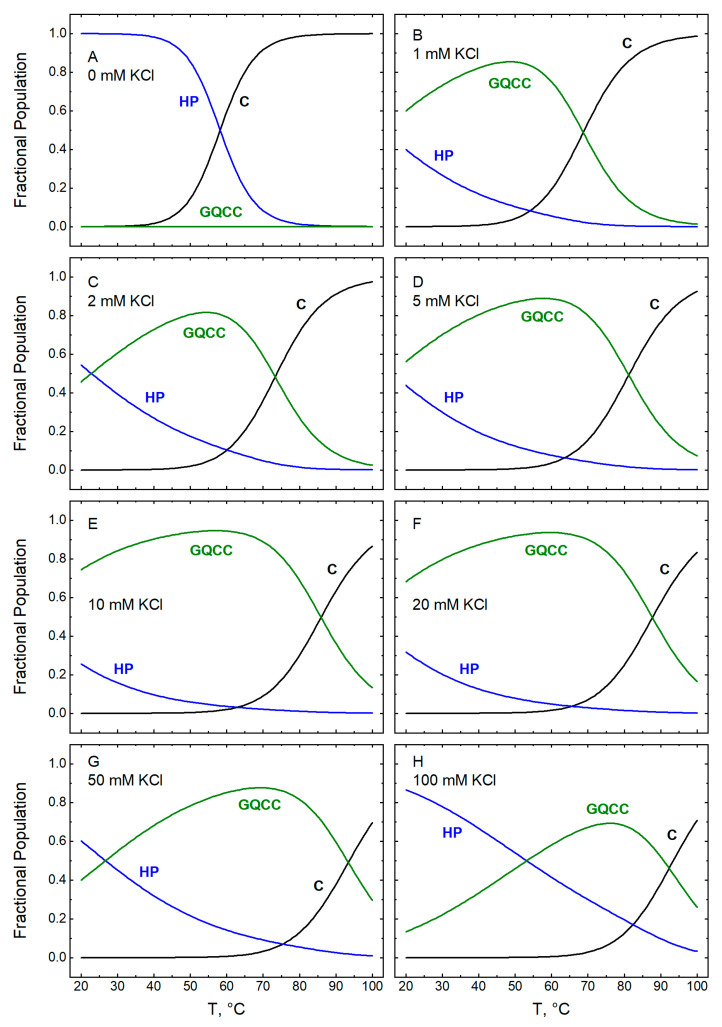
Temperature dependences computed for the fractional populations of three of the five conformational states of GT_11_C (Scheme 1) at pH 7.0: C (black), HP (blue), and GQCC (green). The two *i*-motif-containing states do not exist at pH 7.0 (i.e., iMGC and GQiM). The fractions were calculated according to Equations (6a)–(6e) with the parametric values listed in Table 1 and Table 3 for the following concentrations of KCl (mM): 0 (**panel A**), 1 (**panel B**), 2 (**panel C**), 5 (**panel D**), 10 (**panel E**), 20 (**panel F**), 50 (**panel G**), and (**panel H**). Note that the term HP refers to the hairpin state, whereas the term duplex in Figure 11 refers to a secondary structure or conformation. Because the duplex exists only in the hairpin state, the corresponding curves in Figure 11 and Figure 13 are identical.

We now can use Equations (6a)–(6e) in conjunction with the thermodynamic parameters listed in Table 1, Table 2 and Table 3 to calculate the temperature dependences of the fractional populations (α) of the coil state, α_C_, hairpin state, α_HP_, G-quadruplex-plus-C-coil state, α_GQCC_, *i*-motif-plus-G-coil state, α_iMGC_, and G-quadruplex-plus-*i*-motif state, α_GQiM_. Those relationships are shown in Figure 12 (pH 5.0) and Figure 13 (pH 7.0) for the different concentrations of KCl (panels A–H).

### 4.4. Distribution of Conformational States at pH 5.0

At 25 °C, in the absence of KCl, about 60% of the population of GT_11_C exists in the hairpin (HP) state (Figure 12A), while the remaining ~40% adopts the *i*-motif-plus-G-coil (iMGC) state. An increase in the concentration of KCl from 0 to 1 mM gives rise to small amounts of the G-quadruplex-containing state GQiM (cf. Figure 12A,B). A further increase in KCl from 1 to 100 mM is accompanied by a marked increase in GQiM (Figure 12B–H), in accord with the known stabilizing effect of potassium ions on G-quadruplexes [1]. As the temperature increases, the double-tetraplex GQiM state is replaced progressively by the single-tetraplex GQCC state owing to the low thermal stability of the *i*-motif. Ultimately, all folded states melt to the coil state (C).

A closer look at Figure 12B–H reveals that an increase in the fractional population of GQiM (α_GQiM_) at room temperature is observed only between 1 and 50 mM KCl; within this range, α_GQiM_ increases from about 5% to ~50%. Intriguingly, a further increase in the concentration of KCl to 100 mM leads to a decrease in the population of GQiM to ~35% with a concomitant increase in the HP state. A similar effect is even more pronounced at pH 7.0, as discussed below. Whereas the inverse relationship between the populations of the HP and GQiM states suggests that one is achieved at the expense of the other, potassium ions are known to stabilize not only the G-quadruplex conformation (specifically, via interactions within the central cavity) but also the hairpin duplex conformation (nonspecifically, via the polyelectrolyte effect) [60]. Hence, it appears that potassium ions may exert a stronger stabilizing effect on the hairpin than on the G-quadruplex at concentrations of KCl at or above 50 mM.

At all concentrations of KCl, an increase in temperature from 25 °C upwards leads to a complex interplay among all states accessible to GT_11_C at pH 5.0 (Figure 12B–H). The populations of the iMGC and the GQiM states decrease owing to the relatively low thermal stability of the *i*-motif. Melting of the *i*-motif in those states causes an increase in the population of the hairpin duplex, as some of the C-rich strands become incorporated into the duplex, and in the population of the GQCC state. Because the thermal stability of the hairpin duplex is lower than that of the G-quadruplex, the increase in the hairpin population is offset by its temperature-dependent melting; that, in turn, is accompanied by a further increase in the population of the GQCC state as some of the G-strands liberated from the hairpin duplex fold into the G-quadruplex. Finally, at the highest temperatures, the G-quadruplex melts with a concomitant decrease in the population of the GQCC state and an increase in the population of the C state.

### 4.5. Distribution of Conformational States at pH 7.0

In the absence of potassium ions, GT_11_C exists overwhelmingly as a hairpin duplex at pH 7.0 and room temperature (Figure 13A). States that include the *i*-motif (iMGC and GQiM) are absent at any concentration of KCl, consistent with the fact that the formation of an *i*-motif generally requires mildly acidic conditions [51]. At room temperature, an increase in the concentration of KCl from 1 to 10–20 mM leads to an increase in the population of the GQCC state at the expense of the hairpin, consistent with a potassium-induced increase in the stability of the G-quadruplex (Figure 13B–F). A further increase in the concentration of KCl to 50 mM and above leads to a large reduction in the population of GQCC (to ~50% at 50 mM and ~20% at 100 mM KCl) and a concomitant increase in the population of HP (Figure 13G,H). As at pH 5.0, this observation suggests that, at elevated concentrations of potassium ions, nonspecific stabilization of the HP conformation outcompetes the specific stabilization of the GQCC state. As the temperature increases, the HP and the GQCC states both melt to the coiled (C) state at all concentrations of KCl.

### 4.6. Biological Implications

There has been much interest recently in the role of G-quadruplexes and *i*-motifs in transcriptional control [2,6,7,10,12,61,62,63,64,65,66]. Mounting evidence suggests that four-stranded DNA structures serve as enhancers or inhibitors of transcription and that the balance between those effects is determined by the cell cycle and fine-tuned for each gene [42,63]. The formation of G-quadruplexes in the genome must be tightly controlled in a concerted manner by the natural conformational propensities of specific genomic sites and the timely intervention of G-quadruplex-binding proteins [67,68]. Any deviation from the healthy thermodynamic balance between the double-helical and tetrahelical conformations may result in over- or under-expression of the gene, with pathological consequences.

To understand the conformational control of transcription and the modifying role of G-quadruplex-binding proteins, one needs an ability to categorize a genomic site with respect to its propensity to fold into a G-quadruplex or *i*-motif. Such an ability currently does not exist [69]. Indeed, recent works aimed at a genome-wide mapping of G-quadruplexes have revealed that, out of several hundreds of thousands of genomic sites potentially capable of forming G-quadruplexes, only about 1% actually do so [14]. We cannot predict if a particular G- and C-rich DNA segment will form a G-quadruplex or an *i*-motif in competition with or even to the exclusion of the duplex conformation. In this respect, present and previous studies in our laboratory suggest that the answer to this question is not a binary “yes or no”; rather, it is more subtle and involves a redistribution of conformations that co-exist in dynamic equilibrium. The state of that equilibrium depends upon the DNA sequence and environmental conditions [47,48]. Our results also imply that, at some genomic sites, the G-quadruplex conformation may be populated only briefly as the dynamic equilibria between G-quadruplex and duplex are shifted predominantly towards the duplex. This implication may help to explain the puzzling observation that only about 1% of all genomic G-quadruplex sequence motifs form G-quadruplexes [14].

The dearth of quantitative knowledge concerning the outcome of the competition between the prevailing DNA conformation of the genome (B-DNA) and non-canonical conformations (G-quadruplex and *i*-motif) at sites of biological interest is detrimental for the field. First and foremost, it compromises our ability to identify genomic loci that can fold into G-quadruplex and *i*-motif structures that act as elements of control. Also, it hampers efforts to develop a quantitative understanding of the conformational control of genomic events. Finally, it undermines endeavors to target four-stranded structures as sites of therapeutic intervention. Studies involving a broad range of DNA constructs differing in sequence, topology, and molecularity are required to elucidate the much-sought link between the conformational propensities of genomic domains and their biological functions.

Our results are consistent with a model in which the tethered G-rich and C-rich strands of a monomolecular construct can adopt different conformations that co-exist and interconvert spontaneously in a dynamic equilibrium. The tethered complementary stands of GT_11_C may occur in a coiled conformation, or they may fold either singly or together to form a hairpin duplex, a single tetraplex having a G-quadruplex or an *i*-motif, or a double tetraplex with both. This observation echoes our earlier results obtained on bimolecular systems, in which the G-rich and C-rich strands can undergo physical separation [47,48]. Taken together, our studies attest to the possibility that, in many genomic domains rich in guanine and cytosine, canonical and non-canonical DNA conformations coexist in dynamic equilibrium with each other. Moreover, they support and strengthen our hypothesis that transcription could be controlled through such a distribution of conformations and states. In that hypothesis, the level of gene expression is regulated in a thermodynamic manner by fine-tuning the ratio of duplex to G-quadruplex in the G-rich and C-rich regions of a promoter, with a G-quadruplex acting as a steric on- and off-switch or recognition element modulating RNA polymerase activity [47,48].

## 5. Conclusions

The ability to regulate genomic events for therapeutic advantage requires an understanding of the conformational properties of non-canonical DNA. To that end, we designed a hairpin-based DNA construct, GT_11_C, in which complementary G-rich and C-rich strands that replicate the promoter region of the c-MYC oncogene are linked by a dT_11_ loop. The construct can adopt duplex, G-quadruplex, *i*-motif, and coil conformations, and the distribution of conformations sampled under different conditions was characterized by CD spectroscopy in a procedure developed previously in our laboratory [47]. Spectra were recorded at pH 5.0 and 7.0 over a temperature range from 25 to 95 °C and at concentrations of KCl from 0 to 100 mM. The G-quadruplex-containing states GQCC and GQiM are observed only when K^+^ and TBA^+^ ions are both present in TBA^+^-phosphate buffers (pH 7.0) or in TBA^+^-acetate buffers (pH 5.0). In the absence of TBA^+^ ions, GT_11_C adopts either the HP state (at pH 7.0) or a mixture of the HP and iMGC states (at pH 5.0). In a TBA^+^-acetate buffer at pH 5.0, GT_11_C populates all possible conformational states (HP, GQCC, iMGC, GQiM, and C) in proportions that depend upon the temperature and the concentration of KCl. In a TBA^+^-phosphate buffer at pH 7.0, GT_11_C populates only the HP, GQCC, and C states in proportions that similarly depend upon the temperature and the concentration of KCl. Taken together, our present results and those obtained previously on bimolecular DNA systems from the promoter regions of the c-MYC, VEGF, and Bcl-2 oncogenes provide further support for the thermodynamic hypothesis of transcription regulation. That hypothesis, as embodied in a quantitative model, offers a route to the designed regulation of genomic events.

## Figures and Tables

**Table 1:** Transition enthalpies (kcal mol^−1^) ^a^.

pH	ΔH_HP_	ΔH_GQ1_	ΔH_GQ2_	ΔH_iM_
5.0	36.7 ± 1.5	16.5 ± 1.2	12.7 ± 2.3	34.9 ± 1.6
7.0	45.9 ± 2.3	35.2 ± 2.2	ND ^b^	ND ^c^

^a^ Equations (8a)–(8e) were fitted to estimates of the fractional populations of duplex, G-quadruplex, *i*-motif, G-coil, and C-coil obtained at pH 5.0 (Figure 10) or pH 7.0 (Figure 11), with the data at all concentrations of KCl taken together. Single values of ΔH_HP_, ΔH_GQ1_, ΔH_GQ2_, and ΔH_iM_ were common to all the data in each analysis, and the fitted values are listed in the table. Separate values of T_HP_, T_GQ1_, T_GQ2_, and T_iM_ were assigned to the data at each concentration of KCl, and the fitted values are listed in Table 2 (pH 5.0) and Table 3 (pH 7.0). The fitted curves are shown in Figure 10 and Figure 11. ^b^ There is little if any of the GQiM state at pH 7.0. The values of T_GQ2_ and ΔH_GQ2_ therefore were set equal to T_GQ1_ and ΔH_GQ1_, respectively. ^c^ There is little if any of the iMGC state at pH 7.0. The values of T_iM_ and ΔH_iM_ therefore were undefined and set arbitrarily to −100 °C and 28.7 kcal mol^−1^ (120 kJ mol^−1^), respectively. The constraints were without effect on the sum of squares or the values of other parameters.

**Table 2:** Transition temperatures at pH 5.0 (°C) ^a^.

[KCl], mM	T_HP_	T_GQ1_	T_GQ2_ ^b^	T_iM_
0	45.8 ± 0.9	ND ^c^	ND ^c^	45.9 ± 1.0
1	50.0 ± 0.9	34.7 ± 4.5 ^d^	0 ± 11.4 ^d^	49.0 ± 1.1
2	51.0 ± 0.9	50.9 ± 2.3	29.5 ± 3.6	48.2 ± 1.3
5	55.2 ± 0.9	57.7 ± 2.0	28.7 ± 4.3	50.1 ± 1.5
10	61.3 ± 1.0	70.9 ± 1.7	42.5 ± 5.3	53.4 ± 1.9
20	60.6 ± 1.0	68.9 ± 1.7	48.0 ± 7.2	50.4 ± 2.2
50	62.4 ± 1.1	76.7 ± 1.6	58.8 ± 9.7	51.5 ± 2.7
100	67.0 ± 1.0	80.0 ± 1.7	53.3 ± 10.3	50.2 ± 3.0

^a^ Equations (8a)–(8e) were fitted to estimates of the fractional populations of duplex, G-quadruplex, *i*-motif, G-coil, and C-coil obtained at pH 5.0 and different concentrations of KCl (Figure 10). The fitted values of T_HP_, T_GQ1_, T_GQ2_, and T_iM_ are listed in the table. Further details are described in the legend to Table 1. ^b^ Values of T_GQ2_ are defined by shallow minima in the sum of squares. Estimates of the associated error therefore are comparatively large. ^c^ There is little or no G-quadruplex at 0 mM KCl. The values of T_GQ1_ and T_GQ2_ therefore were undefined and set arbitrarily to −100 °C. The constraints were without effect on the sum of squares or the values of other parameters. ^d^ Amounts of G-quadruplex at 1 mM KCl are significantly greater than zero (*p* = 0.0061) but small nonetheless (Figure 10B), and the estimates of T_GQ1_ and T_GQ2_ are qualified accordingly.

**Table 3:** Transition temperatures at pH 7.0 (°C) ^a^.

[KCl], mM	T_HP_	TG_Q1_	T_GQ2_ ^b^	T_iM_ ^c^
0	58.2 ± 0.8	ND	ND	ND
1	54.1 ± 1.3	68.7 ± 1.1	ND	ND
2	60.1 ± 1.2	73.3 ± 1.2	ND	ND
5	63.6 ± 1.3	81.2 ± 1.2	ND	ND
10	62.8 ± 1.6	85.9 ± 1.2	ND	ND
20	65.5 ± 1.5	87.7 ± 1.2	ND	ND
50	75.4 ± 1.4	93.4 ± 1.4	ND	ND
100	82.4 ± 1.3	92.3 ± 1.4	ND	ND

^a^ Equations (8a)–(8e) were fitted to estimates of the fractional populations of duplex, G-quadruplex, *i*-motif, G-coil, and C-coil obtained at pH 7.0 and different concentrations of KCl (Figure 11). The fitted values of T_HP_, T_GQ1_, T_GQ2_, and T_iM_ are listed in the table. Further details are described in the legend to Table 1. ^b^ There is very little if any of the GQiM state at pH 7.0. The values of T_GQ2_ and DH_GQ2_ therefore were set equal to T_GQ1_ and DH_GQ1_, respectively. ^c^ There is very little if any of the iMGC state at pH 7.0. The values of T_iM_ and DH_iM_ therefore were undefined and set arbitrarily to −100 °C and 28.7 kcal mol^−1^ (120 kJ mol^−1^), respectively. The constraints were without effect on the sum of squares or the values of other parameters.

## Data Availability

Data are contained within the article.
